# Nanostructured Materials for Carbon Neutrality

**DOI:** 10.3390/nano15030192

**Published:** 2025-01-26

**Authors:** Yan Zhao, Qiu He

**Affiliations:** 1 College of Material Science and Engineering, Sichuan University, Chengdu 610065, China; hq5220@scu.edu.cn; 2 The Institute of Technological Sciences, Wuhan University, Wuhan 430072, China

In the face of escalating climate change due to excessive fossil fuel consumption, the urgency of addressing global warming has never been more critical. The increasing frequency of extreme weather events—droughts, floods, heat waves, heavy rain, and landslides—along with rising sea levels, ocean acidification, and biodiversity loss, underscores the pressing need for action. To limit global warming to 1.5 degrees Celsius, it is essential to achieve carbon neutrality by the mid-21st century, as outlined in the Paris Agreement signed by 195 countries.

This Special Issue of *Nanomaterials* focuses on nanostructured materials for carbon neutrality, highlighting innovative approaches in the design, synthesis, and theoretical studies of nanostructured materials. We particularly welcomed interdisciplinary work that bridges basic science and engineering disciplines.

Key areas of interest include the following:

1. Novel carbon-neutral technologies to reduce greenhouse gas emissions.
2. Renewable energy technologies to replace fossil fuel consumption.
3. Carbon capture, storage, and utilization.
4. Electrocatalytic and photocatalytic CO_2_ reduction reactions (CO_2_RRs).
5. Novel nitrogen reduction reactions (NRRs) at ambient conditions, alongside reactions for oxygen reduction (ORRs), oxygen evolution (OERs), and hydrogen evolution (HERs).
6. Rechargeable battery materials, including electrolytes, electrodes, and separators.

The research highlights are summarized in the following:

1. Hierarchical ZSM-5 zeolite for propane aromatization: Zhang et al. [1] presented an innovative method using low-cost disinfectants to synthesize ZSM-5 zeolite with enhanced catalytic performance. The use of methyltriphenylphosphonium bromide (MTBBP) yields a nano-sized hierarchical ZSM-5 with a “rice crust” morphology, showing better catalytic performance in propane aromatization. The catalyst exhibits a prolonged lifetime and enhanced total turnover number compared to conventional ZSM-5.
2. Nanomaterials for potassium storage: There are two papers dedicated to potassium battery materials. Liu et al. [2] designed a carbon composite anode by confining short-chain sulfur in nitrogen-doped carbon nanospheres. The high content of short-chain sulfur and nitrogen ensures sufficient active sites for K^+^ storage. The electrode exhibits a high reversible capacity of 472.05 mAh g^−1^ at 0.1 A g^−1^ and good rate capability (172 mAh g^−1^ at 2 A g^−1^). The special hollow structure provides ample space for sulfur reactants and effectively mitigates volume expansion during the sulfur conversion process. In another study, Mu et al. [3] developed an anode material based on porous carbon nanosheet/Cu_2_S composites using a self-template method and vulcanization process. The composites exhibit good rate and cycle performance as anode materials for K^+^ batteries, with capacities of 363 mAh g^−1^ at 0.1 A g^−1^ after 100 cycles and 120 mAh g^−1^ at 5 A g^−1^ after 1000 cycles. This research underscores the importance of structural design in enhancing battery efficiency and longevity.
3. Solid oxide fuel cells (SOFCs): Jiang et al. [4] developed a Mo-doped La_0.6_Sr_0.4_Fe_0.8_Ni_0.2_O_3−δ_ material for use as a symmetrical electrode in direct-ammonia solid oxide fuel cells (DA-SOFCs). The material exhibits excellent catalytic activity for ammonia decomposition and high oxygen reduction reactivity, delivering a peak power density of 487 mW cm^−2^ at 800 °C. The cell shows strong operational stability over 400 h at 700 °C, which marks a significant advancement in fuel cell technology. With excellent catalytic activity and operational stability, this work highlights the potential of ammonia as a sustainable fuel source.
4. Carbon nanosheets for CO_2_ reduction: Bai et al. [5] reported the fabrication of a novel photocatalyst by growing Bi_2_MoO_6_ nanosheets on 3D N,O-doped carbon (NO-C) nanosheets. The structure enhances the contact between Bi_2_MoO_6_ and NO-C, reducing the stacking of NO-C layers and providing channels for CO_2_ diffusion. The catalyst shows excellent performance in photocatalytic CO_2_ reduction, with methane and CO yields of 9.14 and 14.49 μmol g^−1^ h^−1^, respectively. The unique 3D structure and synergistic effect contribute to its high activity and stability.

In summary, this Special Issue, “Nanostructured Materials for Carbon Neutrality”, reflects the diversity and creativity of new nanomaterials and fabrication processes addressing the urgent need for carbon neutrality. We hope this Special Issue inspires groundbreaking research and fosters collaboration across disciplines, driving progress toward a sustainable, carbon-neutral future.
